# A New Manual Alternative to the Ober Test: A Pilot Study of Precision and Accuracy in Healthy Young Adults

**DOI:** 10.7759/cureus.95138

**Published:** 2025-10-22

**Authors:** Corrado Borghi, Saverio Colonna, Alejandro Roldan Garcia

**Affiliations:** 1 Ostheopathic Spine Center Education, Spine Center, Bologna, ITA; 2 Rehabilitation Medicine, Spine Center, Bologna, ITA; 3 Research and Development, Osteopathic Spine Center Education, Bologna, ITA

**Keywords:** hip abductor myofascial assessment, hip joint, iliotibial band, iliotibial band tightness, manual examination technique, myofascial pain syndromes, ober test, ober test alternative, physical examination, range of motion

## Abstract

The objective of this study was to evaluate the intra-rater and inter-rater reliability of a new clinical test for assessing the hip adductor myofascial system and determine its accuracy by comparison with inertial sensor data.

A diagnostic reliability study was conducted involving 46 healthy participants (92 limbs) aged 18-30 years. Each participant underwent a standardized assessment using a newly developed manual test for hip adduction, performed by two trained examiners during two separate sessions (T0 and T1). Inertial sensors were used to measure hip adduction angles as the reference standard. Intra-rater and inter-rater reliability were assessed using intraclass correlation coefficients (ICC). Agreement between visual assessments and instrumented measurements was also analyzed. Post-hoc power calculations were performed for all ICC values.

The new test demonstrated good intra-rater reliability, with ICC values of 0.881 for examiner 1 and 0.840 for examiner 2. Inter-rater reliability was also good, with ICC values of 0.888 at T0 and 0.886 at T1. Agreement between visual assessments and inertial sensor measurements was high, with ICC values of 0.898 for examiner 1 and 0.931 for examiner 2. Bland-Altman plots confirmed strong agreement across all comparisons, and power analyses showed sufficient sample size for all ICC calculations.

The new test provides a reliable and accurate assessment of hip adductor myofascial extensibility, addressing key limitations of the Ober test. Its good intra- and inter-rater reliability, along with ease of use in clinical settings without instrumentation, make it a promising alternative. Future research should explore its application across diverse populations and its clinical correlation with iliotibial band-related pathologies.

## Introduction

The iliotibial band (ITB), also known as the iliotibial tract or Maissiat's band, is a fibrous fascial tissue that reinforces the fascia lata laterally to the thigh [1-3]. Biomechanically significant, the ITB aids in maintaining an upright posture, stabilizing the knee, and facilitating movements of the gluteus maximus and tensor fascia lata muscles [4,5]. When the ITB is in a state of incorrect tension, it can easily become pathologically affected, leading to conditions such as ITB syndrome [5-8].

ITB syndrome is characterized by pain on the lateral side of the knee, often occurring during activities such as running, especially downhill, cycling, and other sports involving repetitive knee flexion and extension [8-11]. Given its prevalence among athletes, particularly runners, accurate assessment of ITB tension and flexibility is crucial for both diagnosis and treatment.

In addition to ITB syndrome, other conditions have been linked to ITB tension. A 2022 study [12] found that 21.3% of 91 patients with anterior cruciate ligament injuries also had deep ITB lesions. Conversely, a 2010 study [13] found no correlation between low back pain and ITB stiffness but emphasized the need for further research on the imbalance between hip abductor muscle weakness and ITB stiffness in patients with low back pain.

The Ober test, introduced in 1937, is the main clinical method used to evaluate ITB extensibility. In the classic version, the patient lies on the side with the lower leg flexed, and the therapist lets the upper leg drop toward the table from a 90° knee-flexed position. If the ITB is tight or rigid, the leg will remain stable (positive test); otherwise, it will fall due to gravity. The modified Ober test differs in that the patient’s upper leg remains extended at the knee throughout the procedure, aiming to minimize the influence of knee flexors and patellar tension [14]. The modified Ober test is considered positive when the patient's leg does not drop down to the table or horizontal plane.

Both versions of the Ober test have significant limitations. The classic Ober test allows for excessive freedom of pelvic movement and femur rotation, which can affect the accuracy of the assessment [14-16]. It also lacks quantitative data, as it relies on the therapist's visual estimation rather than precise measurements [16,17]. The modified Ober test attempts to address some of these issues by keeping the knee extended, but it still suffers from similar problems, including potential pelvic and femoral rotation, and the qualitative nature of its results. Additionally, neither version of the Ober test has undergone extensive validation for reliability or accuracy [16,18,19].

Continuing to assess ITB extensibility in this approximate manner will not clarify why, from a biomechanical and clinical standpoint, correlations have been found between the ITB and pathological conditions like those previously mentioned, while at the same time, literature has not found a correlation between ITB tightness, as evaluated by the classic and modified Ober tests, and these same conditions [13,18,19].

Some authors have attempted to make this test more precise and reliable. In two studies [15,20], a goniometer was used to assess hip adduction. In another study [17], a tape measure was used to record the linear distance between the patella and the support plane. In two other studies, an inclinometer placed on the lateral aspect of the evaluated thigh was used [14,16].

The alternative of always using instrumental measurement (e.g., with motion sensors) could provide accurate and reliable data but would require excessive time, cost, and expertise for daily use in clinical practice [21].

Given these limitations, there is a clear need for a new, more reliable test that can provide accurate, reproducible measurements of ITB tightness. This study aims to introduce and validate a new test designed to address the weaknesses of both the classic and modified Ober tests.

## Materials and methods

Study design

This study was designed to evaluate the reliability and accuracy of a new test for assessing ITB tightness. The primary outcomes were the intra-rater and inter-rater reliability of the new test, measured using the intraclass correlation coefficient (ICC). Secondary outcomes included the accuracy of the new test compared to an inertial motion sensor and the amount of pelvic movement observed during the new test versus the modified Ober test.

Measurement instruments

The angles of the tested leg were measured with inertial sensors (Xsens®, Xsens Technologies B.V., Enschede, The Netherlands), which are commercially available devices, employed under institutional license. Their reliability and concurrent validity in clinical movement analysis have been previously reported [21]. The device was handheld and aligned with the adduction vector; contact with the distal leg was cushioned by a strap to avoid discomfort. The outcome of interest remained the hip adduction angle in degrees, as visually assessed and verified against the inertial motion sensors, consistent with our original description. A custom-made frame (lower limb simulator) was used exclusively during examiner training to rehearse a consistent trajectory and direction of pull, in combination with angular markers and inertial-sensor feedback. The frame is not a measuring instrument, not patented (not subject to any licensing requirements), and was not used during formal data collection or analysis. Its purpose is educational standardization (force awareness and vector alignment).

We used a commercial handheld dynamometer (Kern®, Kern & Sohn GmbH, Balingen, Germany) to standardize the traction force applied to the lower limb simulator. During training, the handheld dynamometer was mounted inline on the lower limb simulator, between the arm and the traction strap, providing real-time force feedback to reach 100 N; it was not attached to the participant, and no dynamometer readings were used as outcome data. It was used only to standardize force, not to derive outcome measures. This device is commercially available and does not require a specific license for clinical or research use.

Examiners also used a standard goniometer and angular markers on the examination table to visually identify hip adduction angles. These tools are freely available, non-proprietary instruments commonly used in clinical and research settings. The outcome measure was the hip adduction angle, expressed in degrees, which is a universally adopted and license-free unit of measurement.

Participants

The only exclusion criterion was the absence of symptomatic lower limb conditions that could impede measurement, such as ligament injuries of the knee or degenerative joint diseases of the hip. It was deemed unnecessary and arbitrary to adopt any further restrictions or specifications. In fact, there is no existing research on ITB tightness that categorizes subjects by characteristics such as age, sex, or pathology.

A total of 40 participants (20 males and 20 females, for a total of 80 lower limbs) aged between 18 and 30 years were required to complete two assessment sessions.

In case of drop-out between sessions, additional recruitment was planned to ensure the minimum number of participants was maintained. This sample size was chosen following the guidelines provided by the International Academy of Manual/Musculoskeletal Medicine [22].

Ethical approval for this study was obtained from the IRB Manus Sapiens, approval no. 2022_003. All procedures complied with relevant regulations, and informed consent was obtained from all participants.

New test execution

This new test was proposed in the literature by Colonna [23]. It was designed to provide a more accurate and reliable measurement by minimizing pelvic movement and offering a clearer assessment of hip adduction. The test is performed as follows:

1. Patient positioning: The patient lies supine on the edge of a treatment table. The leg to be tested is extended alongside the edge. The leg that is not being tested is flexed at both the hip and knee, with the lateral malleolus resting against the table edge. This position helps stabilize the pelvis and minimizes any unwanted pelvic tilt or rotation during the test.

2. Examiner positioning: The examiner stands on the opposite side of the leg to be tested. One hand is used to stabilize the knee of the non-tested leg, maintaining its position against the table, which further ensures that the pelvis remains stable. The other hand grasps the ankle of the leg to be tested.

3. Hip adduction: The examiner then adducts the tested leg by gently guiding it onto the surface of the table, ensuring that the movement does not cause any pain to the patient. The leg is adducted until the examiner feels a determined resistance or the patient indicates discomfort.

4. Measurement: The angle of hip adduction is then visually assessed, assigning a value in degrees. This angle represents, by extension, the extensibility of the ITB.

**Figure 1:**
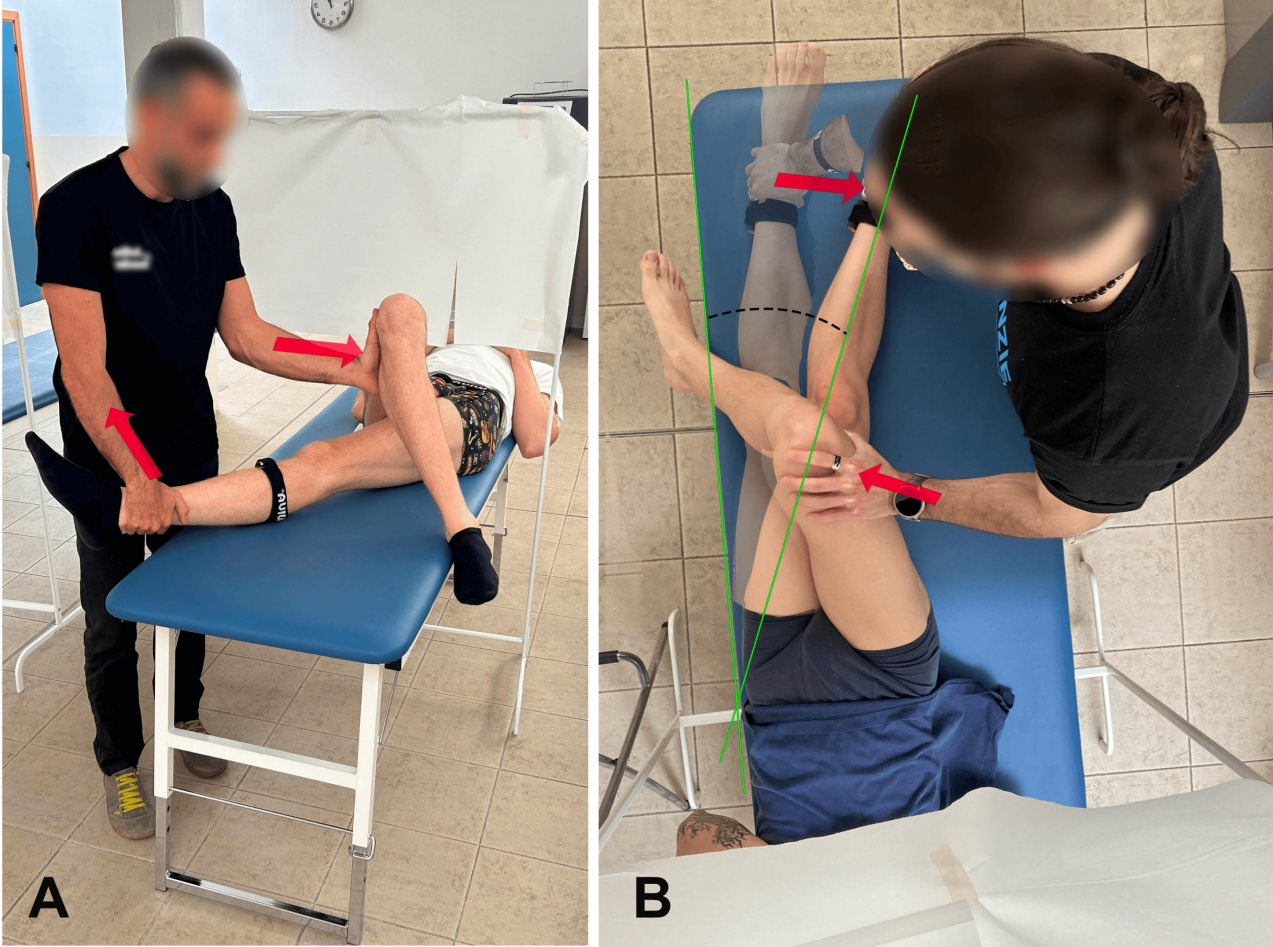
Examiners 1 and 2 performing the new hip abductor tightness test. (A) Caudal view; (B) top view. Image credit: Corrado Borghi.

This new test aims to reduce the potential for errors present in other tests, such as the modified Ober test, by providing a more controlled environment where extraneous movements are minimized, allowing for a more precise measurement of hip adduction. Furthermore, the position of the examiner's eyes relative to the edge of the treatment table serves as an optimal reference point, enhancing the consistency and accuracy of the hip angle evaluation (Figure 1).

Phases of the study

**Figure 2:**
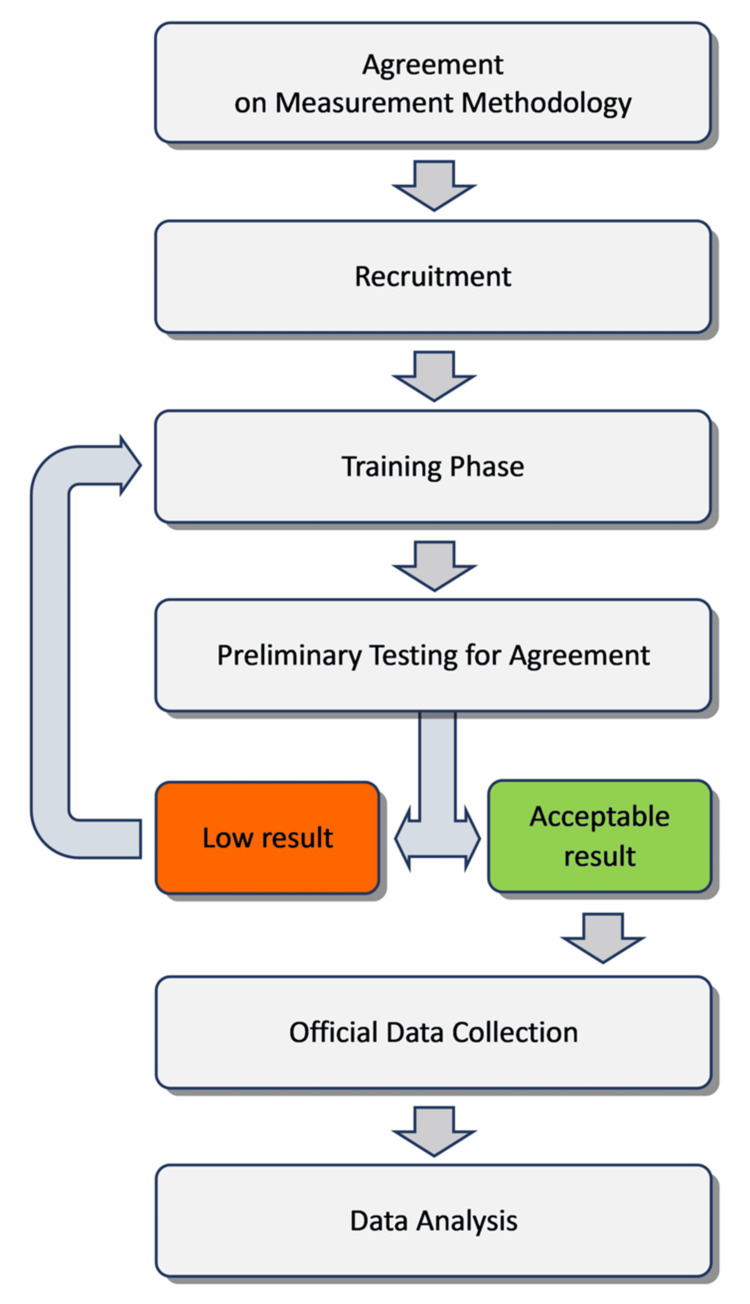
Flowchart of study phases. Flowchart illustrating the different phases of the study, from agreement on the measurement methodology to recruitment, training, preliminary testing for agreement, and subsequent data collection and analysis. Image credit: Corrado Borghi.

The study was conducted in six phases (Figure 2).

Agreement on Measurement Methodology

Initial discussions were held to standardize the procedure for both the new test and the modified Ober test, ensuring consistency in test execution. This phase required approximately four hours to complete.

Recruitment

Forty participants were recruited according to the inclusion and exclusion criteria at the Osteopathic Spine Center Education (OSCE) school of osteopathy in Bologna, Italy, through announcements during the lessons and on the bulletin board.

Training Phase

**Figure 3:**
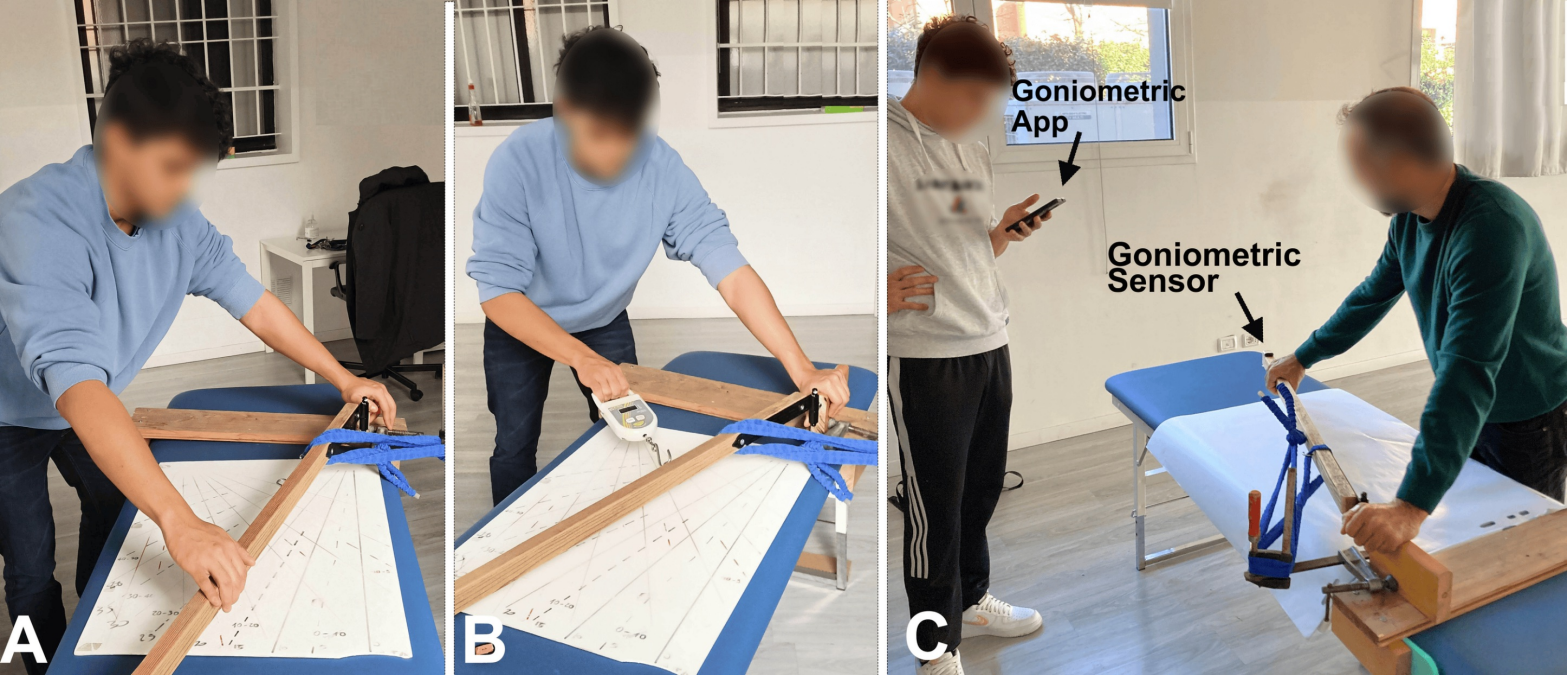
Examiner training phases. (A) Training with the lower limb simulator and angular markers on the examination table to visually identify angles. (B) Training with the simulator and a dynamometer to develop awareness of applied force. (C) Training with the simulator and an inertial sensor to verify angle accuracy, with real-time feedback provided via a dedicated mobile app controlled by a second examiner. Image credit: Corrado Borghi.

The training phase aimed to ensure that examiners performed the tests consistently and accurately, using a custom-made device specifically designed to reproduce the hip adduction movement (Figure 3).

Before formal data collection, examiners practiced the maneuver using angular markers drawn on the examination table to calibrate visual estimation of adduction angles (Figure 3A); the handheld dynamometer to develop awareness and consistency of the applied force (100 N) (Figure 3B), and inertial sensors for real-time feedback (Figure 3C).

To facilitate the training, we used a lower limb simulator positioned over the table: this training aid provided fixed reference points for hand/strap placement and direction of pull, helping examiners rehearse a consistent adduction path. The frame carries no measurement function, was not used during outcome acquisition, and serves purely as an educational standardization tool. 

Examiners practiced the new test on volunteers using a goniometer to visually assess hip adduction angles (Figure 1). They also used a dynamometer (Figure 3B) to standardize the force applied during the test (which was set at 100N). Examiners were trained to recognize and minimize pelvic movement to improve test reliability. They practiced using inertial sensors to validate their visual assessments and received feedback on their performance to refine their technique.

Preliminary Testing for Agreement

Examiners conducted preliminary tests on a subset of 10 participants to assess the consistency of their measurements and refine their technique further. The minimum threshold for the ICC to be considered acceptable was set at 0.6, according to Patijn [22].

Official Data Collection

Each examiner conducted the new test on both lower limbs of each participant twice (at T0 and T1), with a minimum interval of four hours between assessments. During this interval, participants were instructed to refrain from engaging in any activities that could alter the tightness of the hip adductors.

Data Analysis

The collected data were analyzed to determine the reliability and accuracy of the new test compared to the inertial sensor measurements.

Statistical analysis

Intra-rater and inter-rater reliability were assessed using the ICC, interpreted as follows: values below 0.50 indicate poor reliability, between 0.50 and 0.75 moderate reliability, between 0.75 and 0.90 good reliability, and above 0.90 excellent reliability [24]. The ICC was also used to assess agreement between the manual test and the inertial sensor measurements. To visualize the level of agreement, Bland-Altman plots were generated for each comparison, illustrating the mean differences and 95% limits of agreement. A post hoc power analysis was performed for each ICC comparison to evaluate the statistical adequacy of the sample size. The analysis was based on the z-transformation approach for ICC testing, assuming a minimum acceptable ICC of 0.60. Power was calculated using the formulation described by Walter et al. [25], with the number of independent observations corresponding to the number of subjects (not limbs), as the two lower limbs of the same individual cannot be considered statistically independent.

## Results

To assess the reproducibility of the observations, the ICC was used, which is useful for evaluating the consistency and reproducibility of quantitative measurements made by different examiners (or at different times by the same examiner) for the same phenomenon. The statistical unit for the described analyses is the evaluation of the individual limb. After the first 10 subjects tested (for a total of 20 evaluations per examiner), the ICC between the two examiners was 0.72. This value was considered acceptable, as it exceeded the defined threshold of 0.6, allowing the evaluations to proceed [22].

After the measurements at T0 were completed, 6 of the 40 subjects tested communicated that they would not be available for T1, when the retests were to be conducted. Therefore, it was necessary to recruit 6 additional subjects to ensure that 40 measurements could also be performed at T1. This resulted in a total of 46 subjects (age 23 ± 4 years; weight 67 ± 12 kg; height 171 ± 9 cm) tested at T0 and 40 subjects tested at T1. The data from the 6 subjects who were only present at T0 were still recorded and used where applicable. Across the full sample, the mean hip adduction angle measured with inertial sensors was 31.2° (standard deviation (SD) = 8.6°); subgroup analysis revealed a mean of 27.7° (SD = 8.4°) in males (*n* = 22) and 34.4° (SD = 7.6°) in females (*n* = 24).

Examiner-instrument agreement

For the evaluation of the agreement between the examiners and the instrument, the 92 measurements (46 subjects, two limbs each) taken at T0 by both examiners were considered (Table 1).

**Table 1:** Examiner-instrument agreement. Descriptive statistics (mean and standard deviation (SD)), intraclass correlation coefficients (ICC), and post hoc power values for the agreement between each examiner and the corresponding inertial sensor. Power was calculated using the z-based method proposed by Walter et al. [25], with a minimum acceptable ICC of 0.60 and a sample size of 46 subjects (*N*).

	Mean (SD) (°)	N	ICC	Power
Sensor 1 (T0)	30.4 (8.8)	46	0.90	>0.99
Examiner 1 (T0)	30.9 (8.2)			
Sensor 2 (T0)	32.0 (8.4)	46	0.93	>0.99
Examiner 2 (T0)	31.3 (8.1)			

The ICC obtained for examiner 1, with a 95% confidence interval, was ICC1X = 0.898 (0.850; 0.932) 95%. The observed probability for examiner 1 of not having reproducibility ability is 4.7 × 10^-32^%. The ICC obtained for examiner 2 was ICC2X = 0.931 (0.898; 0.954) 95%. The observed probability for examiner 2 of not having reproducibility ability was 1.89 × 10^-39^%.

The results for examiner 1 demonstrated good agreement with the inertial sensors, while those for examiner 2 showed excellent agreement. In both cases, the null hypothesis of no reproducibility was rejected, but perfect reproducibility was also excluded at a 95% confidence level.

The average absolute difference between the visual assessments and the sensor readings was 2.5 degrees.

Intra-examiner agreement

For the evaluation of intra-examiner agreement, 80 measurements taken by both examiners were considered (only the subjects present both at T0 and T1 were evaluated; Table 2).

**Table 2:** Intra-examiner agreement. Descriptive statistics (mean and standard deviation (SD)), intraclass correlation coefficients (ICC), and post hoc power values for the test/re-test agreement of the same examiner. Power was calculated using the z-based method proposed by Walter et al. [25], with a minimum acceptable ICC of 0.60 and a sample size of 40 subjects (*N*).

	Mean (SD) (°)	N	ICC	Power
Examiner 1 (T0)	31.1 (7.8)	40	0.91	>0.99
Examiner 1 (T1)	33.2 (7.1)			
Examiner 2 (T0)	31.4 (7.3)	40	0.86	>0.99
Examiner 2 (T1)	32.6 (7.5)			

The ICC obtained for examiner 1, with a 95% confidence interval, was ICC1 = 0.881 (0.808; 0.925) 95%. The observed probability for examiner 1 of not having reproducibility ability was 7.3 × 10^-18^%. The ICC obtained for examiner 2 was ICC2 = 0.840 (0.663; 0.915) 95%. The observed probability for examiner 2 of not having reproducibility ability was 1.66 × 10^-5^%. Again, in both cases, the null hypothesis of no reproducibility was rejected.

Inter-examiner agreement

For the evaluation of inter-examiner agreement, the 92 measurements taken during the first evaluation (T0; two limbs for 46 subjects) and the 80 measurements taken during the second evaluation (T1; two limbs for 40 subjects) by both examiners were considered (Table 3).

**Table 3:** Inter-examiner agreement Descriptive statistics (mean and standard deviation (SD)), intraclass correlation coefficients (ICC), and post hoc power values for the agreement between the two examiners. Power was calculated using the z-based method proposed by Walter et al. [25], with a minimum acceptable ICC of 0.60 and a sample size of 46 subjects (*N*) at T0 and 40 subjects at T1.

	Mean (SD) (°)	N	ICC	Power
Examiner 1 (T0)	30.9 (8.2)	46	0.79	>0.99
Examiner 2 (T0)	31.3 (8.1)			
Examiner 1 (T1)	33.2 (7.1)	40	0.81	>0.99
Examiner 2 (T1)	32.6 (7.5)			

The ICC obtained at T0, with a 95% confidence interval, was ICCT0 = 0.888 (0.836; 0.925) 95%. The observed probability at T0 of not having reproducibility ability was 4.73 × 10^-31^%. The ICC obtained at T1 was ICCT1 = 0.886 (0.828; 0.926) 95%. The observed probability at T1 of not having reproducibility ability was 2.97 × 10^-26^%. These results demonstrated good reproducibility, excluding the null hypothesis of no reproducibility.

Consistency of the applied force

This analysis represents a subset of the inter-examiner repeatability assessment previously described. While inter-examiner reliability evaluates the overall agreement between different examiners, this specific analysis focuses on whether both examiners exerted a comparable force during testing, as inferred from the agreement of hip adduction angles recorded by the Xsens system: since the sensor measurements are primarily influenced by the traction force exerted on the tested limb rather than by visual observation, a higher agreement in inertial sensors values between the two examiners suggests a greater consistency in the applied force.

To assess this, 92 measurements obtained with the inertial sensors from both examiners were analyzed (Table 4). The correlation coefficient was Rho_x1,2_ = 90.22% (85.55%; 93.44%) 95%, indicating a strong correlation while excluding the possibility of perfect agreement at a 95% confidence level. The ICC for the sensors' measurements between the two examiners was ICC_x1,2_ = 0.886 (0.803; 0.931) 95%, confirming good reproducibility while ruling out perfect agreement.

**Table 4:** . Inter-sensors agreement. Descriptive statistics (mean and standard deviation (SD)), intraclass correlation coefficients (ICC), and post hoc power values for the agreement between the inertial sensors. Power was calculated using the z-based method proposed by Walter et al. [25], with a minimum acceptable ICC of 0.60 and a sample size of 46 subjects (*N*) at T0.

	Mean (SD) (°)	N	ICC	Power
Sensor 1 (T0)	30.4 (8.8)	46	0.76	>0.99
Sensor 2 (T0)	32.0 (8.4)			

The probability that the two examiners lacked agreement in sensor-recorded data was extremely low (2.1 × 10⁻¹³%). These findings confirm that, at a 95% confidence level, the examiners demonstrated a significant level of consistency in the force applied during testing.

**Figure 4:**
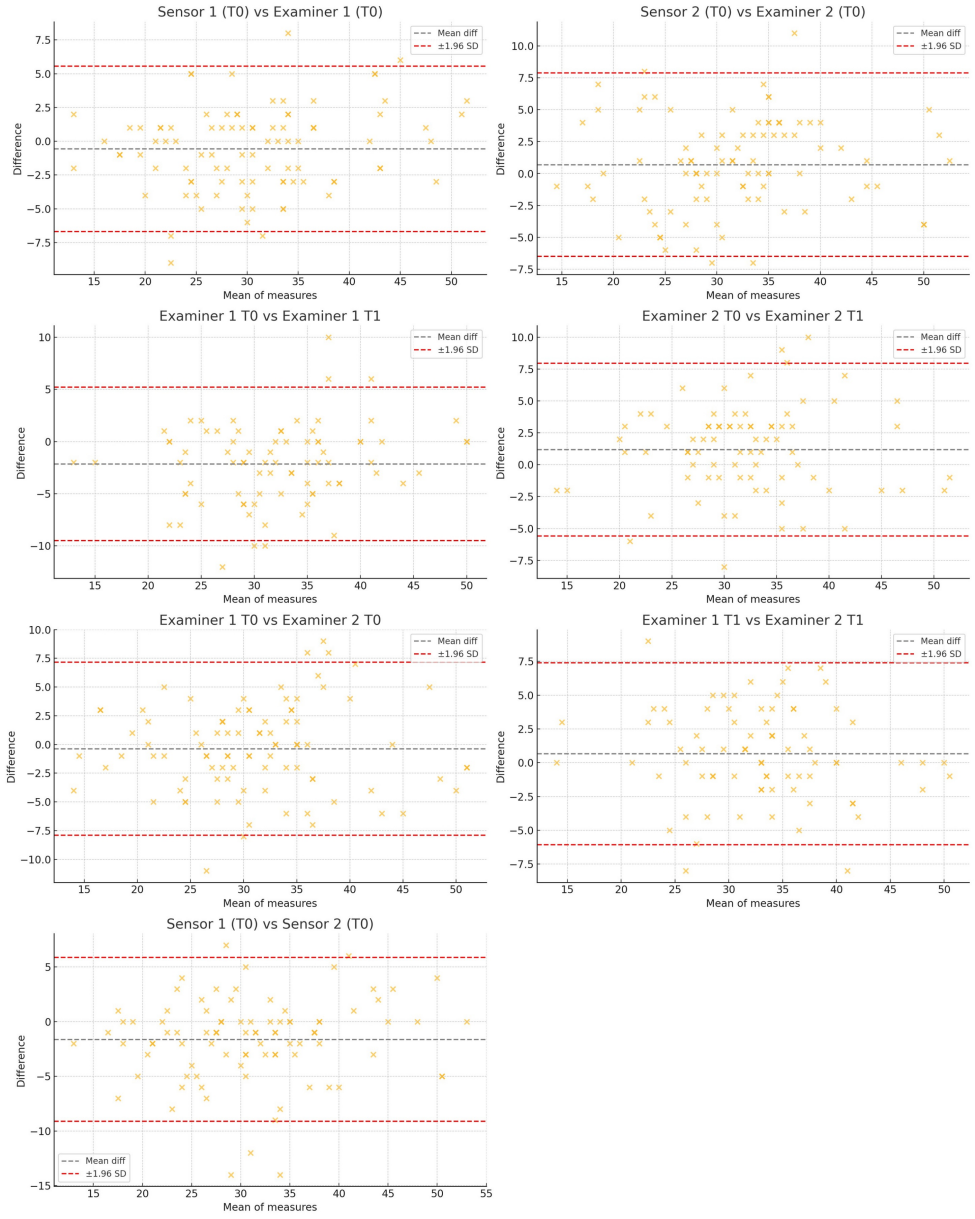
Bland-Altman plots showing agreement between manual and instrument-based assessments, as well as between raters. Each plot displays the mean difference and the 95% limits of agreement (±1.96 SD) between two methods or raters. Comparisons include sensor 1 vs. examiner 1, sensor 2 vs. examiner 2, examiner 1 vs. examiner 2 (at T0 and T1), sensor 1 vs. sensor 2, and intra-rater comparisons for examiner 1 and examiner 2 (T0 vs. T1). The plots show good consistency across measurements, with no substantial bias or heteroscedasticity observed.

Agreement between the different measurements was further explored using Bland-Altman plots, which are presented in Figure 4. These plots illustrate the distribution of differences between paired methods or raters and visually confirm the overall consistency of the new test across sessions, raters, and instrument-based evaluations.

## Discussion

For the new test to be applied effectively in clinical practice, it must meet several essential criteria: accuracy (agreement between the examiner's visual assessment and the instrument’s data), good inter-examiner reproducibility, and good intra-examiner reproducibility.

The agreement between the visual assessments of the examiners and the data recorded by the instrument (ICC1 = 0.898; ICC2 = 0.931) was good for examiner 1 and excellent for examiner 2, as defined by Portney and Watkins [24]. This high level of precision was likely due to the thorough training undertaken by the examiners in preparation for the official measurements. The use of a visual guide (Figure 3), as in this study, allowed the examiners to align their observational assessments of angles with the instrument readings, improving accuracy. The consistent use of the same treatment table across all evaluations also contributed to providing reliable reference points, which helped in identifying specific angles. In addition, the standardized positioning required by the new test (specifically placing the patient supine with the ankle aligned to the edge of the treatment table) helped ensure consistent limb alignment across trials. This fixed anatomical setup reduced compensatory pelvic movements and further enhanced the reliability of both visual and instrument-based angle measurements.

Inter-examiner reproducibility (ICCT0 = 0.888; ICCT1 = 0.886) and intra-examiner reproducibility (ICC1 = 0.881; ICC2 = 0.840) were both classified as good [24]. On average, the difference between the two examiners’ measurements for the same limb was approximately 3°, which is an acceptable margin that is unlikely to affect clinical decisions regarding ITB tightness.

Comparing the values reported for both examiners, it can be concluded that both demonstrated good reproducibility, with examiner 1 showing slightly more consistent results compared to examiner 2. Examiner 1 showed 5% higher reproducibility and narrower confidence intervals than examiner 2.

The good agreement of hip adduction angles recorded by the inertial sensors (ICCX1,2 = 0.886) supports the conclusion that the force exerted by both examiners during the test was consistent. Examiner training played a key role here, with practice sessions and the use of digital dynamometers helping the examiners standardize the force applied when pulling the tested limb, leading to agreement on when each limb had reached its endpoint.

Compared to the modified Ober test, the new test offers a more controlled evaluation environment, particularly regarding pelvic stability.

By positioning the patient supine and stabilizing the contralateral limb, the new test minimizes compensatory pelvic movements that can affect the accuracy of hip adduction measurement. However, the new test introduces additional variables compared to the modified Ober test, such as the need to apply force to the limb (in the Ober test, hip adduction occurs due to gravity) and visually estimating the hip adduction angle (the Ober test's criteria for a positive result are based on whether the limb drops below horizontal).

Importantly, the new test allows for a broader and more continuous scale of values when quantifying dysfunction in the myofascial system, in contrast to traditional orthopedic manual tests that often rely on a dichotomous outcome (e.g., normal vs. pathological). Through proper training, the examiners in this study were able to achieve good agreement on these additional variables, further demonstrating the effectiveness of the new test.

These results are in line with previous intra-rater reliability estimates reported for the modified Ober test, such as those by Reese and Bandy [14], who found an intra-rater ICC of 0.91 using an inclinometer with two examiners, and by Melchione and Sullivan [16], who reported an intra-rater ICC of 0.94 and an inter-rater ICC of 0.73 using a level and inclinometer (but in a sample of only ten subjects).

In comparison, the present study achieved a higher inter-rater ICC (0.888 and 0.886) without requiring measurement instruments or multiple examiners, indicating that the new test may offer a practical and reliable alternative for clinical use. However, this level of reliability was likely supported by the structured training the examiners received, which should be considered in interpreting the generalizability of the results.

Study limitations

This study has several limitations. This was a pilot reproducibility study, and no a priori power analysis was performed. However, our target of 40 participants aligns with discipline guidance for simple reproducibility protocols and with published methodological examples in Manual/Musculoskeletal Medicine. Future confirmatory studies should include a priori power calculations based on prespecified effect sizes (e.g., expected κ).

The population was limited to healthy young adults, which may not reflect the variability seen in a broader clinical population, including older adults or individuals with existing musculoskeletal conditions. For example, the elderly population may have difficulty crossing their legs due to reduced hip mobility. Moreover, while the Xsens system is highly accurate, it is not entirely free from error, and some measurements could have been influenced by factors outside of the examiners' control or the measurement protocol.

## Conclusions

An accurate and reliable assessment of ITB tension is crucial in clinical practice, especially in the management of lower limb and lumbosacral spine conditions. Given the limitations of the Ober test, which lacks validation in terms of intra- and inter-examiner reproducibility and has never been compared to a gold standard, the new test presented in this study aims to fill these gaps. Despite introducing new variables, such as the force applied to the limb and the visual estimation of hip adduction angles, the results of the new test are promising. It demonstrates good reliability and could serve as a viable alternative to the modified Ober test.

The new test has broader applications beyond assessing ITB tension and hip abductors; it can also serve as a tool for future research on the relationship between ITB stiffness and lower limb or lumbosacral pathologies. Despite its reliability, some limitations remain, as with any diagnostic tool. Nevertheless, the new test represents a valuable advancement in clinical practice. Future studies should aim to establish the clinical significance of differences in hip adduction angles and correlate these with specific ITB-related pathologies. Moreover, it will be important to verify the applicability and reliability of the new test across different populations, including various age groups, activity levels, and clinical conditions, to ensure its broader clinical relevance and generalizability.
